# Extracorporeal therapies in the management of paraquat poisoning: a comprehensive review of current evidence

**DOI:** 10.1080/07853890.2026.2621498

**Published:** 2026-01-30

**Authors:** Bo Yang, Dan Ye, Fanzhou Zeng, Zeping Jiang, Hongxian Li, Nanmei Liu

**Affiliations:** a Department of Nephrology, Naval Medical Center of PLA, Naval Medical University, Shanghai, China; b Intensive Care Unit, Naval Medical Center of PLA, Naval Medical University, Shanghai, China

**Keywords:** Paraquat poisoning, extracorporeal therapy, hemoperfusion, continuous renal replacement therapy, therapeutic plasma exchange

## Abstract

**Introduction:**

Paraquat (PQ) poisoning has a high mortality rate due to rapid systemic distribution and oxidative organ damage, particularly in the lungs. Conventional therapies are often inadequate, prompting interest in extracorporeal therapies to enhance PQ clearance.

**Methods:**

This narrative review synthesizes current evidence on hemoperfusion (HP), continuous renal replacement therapy (CRRT), hemodiafiltration (HDF), haemodialysis (HD) and therapeutic plasma exchange (TPE) in PQ poisoning. Studies were evaluated for therapeutic mechanisms, clinical outcomes and optimal implementation strategies.

**Results:**

Early and repeated HP, especially within 4–6 h post-ingestion, improves survival by directly adsorbing PQ. CRRT supports renal function and facilitates sustained toxin removal, with the best outcomes observed when combined with HP. HDF allows prolonged clearance but has limited supporting data. HD, while useful for managing acute kidney injury, appears ineffective for PQ elimination and may increase mortality. TPE shows potential benefits in severe cases if administered early, but evidence is limited to small studies. Combination therapies (e.g. HP with CRRT or HD) demonstrate synergistic effects in improving survival and organ support.

**Conclusions:**

Extracorporeal therapies, particularly early HP and HP combined with CRRT, are promising strategies in PQ poisoning. HD should not be the primary detoxification method but remains important for supportive care. TPE may be beneficial in selected cases. Further randomized trials are needed to optimize treatment protocols and validate combined approaches.

## Introduction

Paraquat (PQ), a highly effective non-selective contact herbicide, is extensively utilized in agriculture worldwide due to its low cost and potent weed-killing properties [1]. Despite its benefits, PQ poses a significant global health threat due to its extreme toxicity to humans following ingestion, dermal exposure, or inhalation [2]. As a small (186 Da), highly water-soluble, poorly lipid-soluble toxin with low protein binding, its physicochemical properties make it a theoretical candidate for removal by extracorporeal therapies [3]. The mortality rate associated with PQ poisoning remains alarmingly high, ranging from 50% to 90% in reported cases, even when relatively small amounts are ingested [4–6]. The ingestion of as little as 5 to 15 millilitres of a 20% aqueous solution can be lethal in adults [7]. The primary mechanism of PQ toxicity involves its intracellular reduction, leading to the generation of highly reactive oxygen species [8,9]. This oxidative stress cascade results in cellular damage through lipid peroxidation, inflammation, apoptosis and ultimately, multi-organ failure. Notably, PQ is actively taken up against a concentration gradient into lung tissue, making the lungs the primary target organ and leading to severe pneumonitis and subsequent fibrosis [10,11]. While early symptoms of PQ intoxication might be non-specific, such as localized corrosive damage or gastrointestinal upset, the disease progresses rapidly, often leading to respiratory failure and death within days to weeks. Diagnosis typically involves a history of exposure, which can be supported by rapid bedside urine dithionite tests and quantitative measurements of plasma PQ levels. The consistently high mortality associated with PQ poisoning, despite various therapeutic interventions, underscores the critical need for effective management strategies, particularly exploring the role of extracorporeal therapies. The rapid and systemic nature of the toxicity necessitates interventions that can swiftly reduce the body burden of the poison and mitigate its devastating effects on vital organs.

Currently, there is no specific antidote available to counteract the toxic effects of PQ. The cornerstone of clinical management relies on supportive care, which includes maintaining airway, breathing and circulation, along with efforts to limit further absorption of the poison through gastrointestinal decontamination methods such as gastric lavage, administration of activated charcoal and Fuller’s earth [12]. Additionally, immunosuppressive therapies, typically involving high-dose glucocorticoids and cyclophosphamide, are frequently employed to reduce inflammation and potentially slow down the progression of pulmonary fibrosis [12]. Antioxidants like vitamin C and N-acetylcysteine are also used to combat the oxidative stress induced by PQ [12]. However, the effectiveness of these conventional treatments remains uncertain, particularly in cases of moderate to severe poisoning, highlighting the ongoing challenge in managing this condition. The absence of a definitive cure underscores the importance of investigating and optimizing supportive and extracorporeal therapies aimed at enhancing the elimination of PQ from the body or mitigating its toxic effects before irreversible damage occurs.

**Figure 1.**
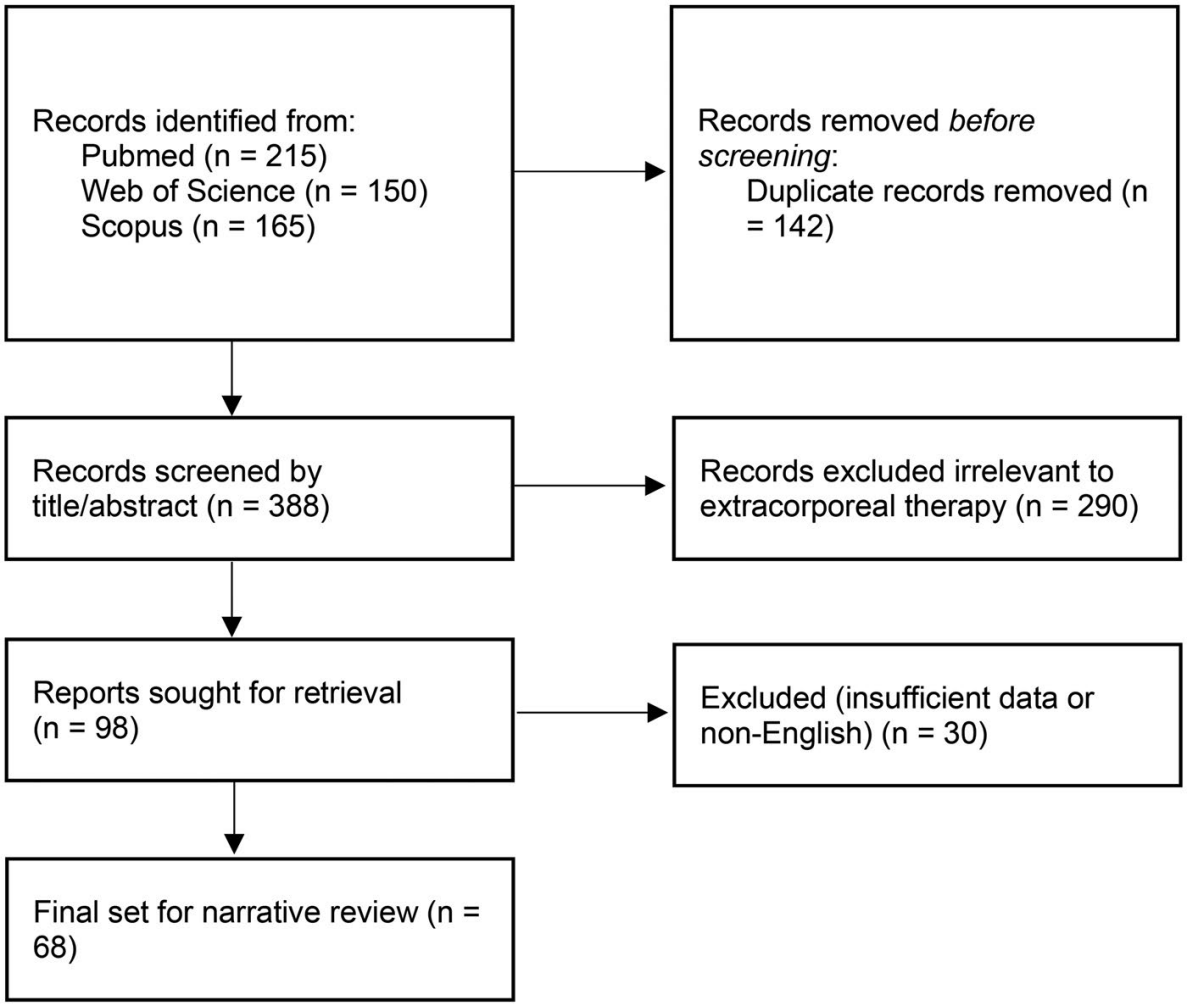
Flow diagram of records included.

Extracorporeal therapies, including hemoperfusion (HP), continuous renal replacement therapy (CRRT), hemodiafiltration (HDF), haemodialysis (HD) and therapeutic plasma exchange (TPE), represent potential modalities for removing toxins from the bloodstream in cases of poisoning [13]. Their application in PQ poisoning is predicated on the rationale of reducing the systemic concentration of the toxin, ideally before it distributes extensively into tissues and causes irreversible organ damage, particularly in the lungs. Given the systemic nature of PQ toxicity and its rapid distribution, the concept of using extracorporeal methods to enhance its elimination is biologically plausible. However, the actual clinical effectiveness of these therapies needs careful and critical evaluation, considering factors such as the timing of intervention relative to ingestion, the extent of PQ distribution into tissues and the overall severity of poisoning. This narrative review article aims to examine the existing evidence regarding the application of HP, CRRT, HDF, HD and TPE in the management of PQ poisoning. To achieve this, a comprehensive literature search was conducted across PubMed, Scopus and Web of Science databases for articles published up to August 2025. Search terms included ‘paraquat’, ‘poisoning’, ‘extracorporeal therapy’, ‘hemoperfusion’, ‘hemodialysis’, ‘hemofiltration’ and ‘plasma exchange’. The review included meta-analyses, clinical trials, observational studies, case series and case reports relevant to the topic (Figure 1). This review synthesizes the findings in a narrative format to provide a broad overview of the current state of knowledge. It will focus on elucidating their mechanisms of action in the context of PQ toxicity, critically appraising the reported clinical outcomes including survival rates, impact on organ function and associated complications, as well as discussing considerations for their optimal utilization in clinical practice (Table 1). A total of 68 articles were ultimately included in this review after screening for relevance to the specific extracorporeal modalities discussed.

**Table 1.** Summary of extracorporeal therapies in paraquat poisoning.

Therapy	Mechanism of action	Evidence of efficacy	Optimal timing	Limitations/complications
Hemoperfusion (HP)	Adsorption of toxins onto activated charcoal or resin	Reduces mortality compared to conventional therapy alone. Combination with other treatments may be more effective.	Early initiation (within 4–6 h) is crucial. Repeated sessions may be beneficial.	Primarily removes macromolecular toxins. Potential for thrombocytopenia, leukopoenia, hypoglycaemia. Efficacy may be limited in very low initial plasma paraquat concentrations.
Continuous renal replacement therapy (CRRT)	Continuous removal of solutes and fluid *via* convection and diffusion	CRRT alone may be better than HP for certain paraquat levels. Combination with HP shows promising results. Supports renal function.	Early stages of poisoning, often within 24 h. Duration typically 8–12 h daily.	Potential for filter clotting when used with hemoperfusion charcoal filters. Limited data in some studies.
Hemodiafiltration (HDF)	Combines haemodialysis (diffusion) and hemofiltration (convection)	Case reports suggest potential in severe poisoning. May allow for longer continuous treatment.	Early initiation is likely beneficial.	Limited evidence compared to HP and HD. Clearance rate might be lower than HP.
Haemodialysis (HD)	Removal of waste and fluid *via* diffusion	Meta-analysis suggests no significant impact on survival, possibly higher mortality. Role primarily in managing acute kidney injury.	Very early initiation might have a supportive role. Typical sessions 2–4 h.	Limited direct paraquat removal due to rapid tissue distribution.
Therapeutic plasma exchange (TPE)	Removal of plasma containing toxins and replacement with fresh plasma or albumin	Case reports and small series suggest potential benefit, especially early.	Early initiation (within 24–36 h in severe cases).	Lack of robust evidence from randomized controlled trials. May be less effective for small, nonprotein-bound molecules. Potential complications like hypotension, allergic reactions, bleeding, sepsis, hypocalcaemia.

## HP in PQ poisoning

HP is an extracorporeal blood purification technique that involves passing the patient’s blood through a cartridge containing adsorbent materials, typically activated charcoal or synthetic resins [14]. The principle of action relies on the adsorption of toxins from the blood onto the surface of these materials [15]. Due to its strong binding affinity for PQ, HP is theoretically well-suited for removing the toxin from the circulation [16]. Early and repeated sessions of HP are thought to be crucial in reducing the overall accumulation of PQ in the body [17]. The direct adsorption mechanism targets circulating PQ, which is critical in the initial hours post-ingestion before the toxin can be sequestered in deep tissue compartments like the lungs, thereby potentially mitigating the development of fatal pulmonary fibrosis. making it potentially most effective in the initial hours following ingestion, before the toxin has extensively distributed into tissues. Factors such as the timing of HP initiation, the amount of PQ ingested, the patient’s baseline clinical status and the use of concurrent therapies (e.g. immunosuppressants, antioxidants) vary widely and can confound outcomes. Therefore, the conflicting results highlight the need for standardized treatment protocols and patient selection criteria in future research.

Evidence from several clinical studies and meta-analyses suggests that HP alone can reduce the odds of death in patients with PQ poisoning when compared to conventional therapy alone [18–20]. One meta-analysis, encompassing a significant number of patients, demonstrated a statistically significant reduction in mortality with HP treatment (relative risk = 0.60; 95% confidence interval [CI] = 0.54–0.66) [18]. Another meta-analysis reported an even more pronounced effect, with an odds ratio (OR) of 0.20 (95% CI: 0.11–0.40, *p* < .0001) for mortality reduction in the HP group compared to those receiving only conventional treatments [19]. However, it is important to note that the benefit of HP as a standalone therapy might be limited, and there is a growing body of evidence suggesting that combining it with other therapeutic modalities could lead to further improvements in patient outcomes [21]. For instance, studies have indicated that patients who received antioxidant or immunosuppressant therapy in addition to HP had better survival rates [22,23]. Conversely, some studies have not found a statistically significant difference in survival between patients treated with HP and those who were not [24]. This inconsistency in findings across studies is likely due to significant heterogeneity in study design and patient populations. The optimal timing for initiating HP appears to be early after PQ ingestion, with many studies emphasizing the importance of starting treatment within the first 4 to 6 h to achieve the best possible results [25,26]. Some research suggests that initiating HP within 12 h of poisoning might also contribute to reduced mortality [22,27]. However, a multi-centre retrospective study found that early HP, defined as the first session performed within 5 h of PQ ingestion, was not significantly related to better survival in their patient cohort [24]. This highlights that while early intervention is generally considered beneficial, the precise optimal window and the impact of ‘very early’ HP might require further clarification. There is also evidence suggesting that repeated sessions of HP could be more beneficial than a single session. One retrospective study identified early, repeated HP, especially when coupled with HD, as a significant factor in improving patient outcomes [17]. In terms of duration, a typical HP session might last around 2 h, with blood flow rates adjusted between 150 to 200 ml/min, and the frequency of ‘repeated’ HP is often highlighted as important to prevent the re-accumulation of toxins.

Several studies have examined the impact of HP on survival outcomes and organ function in PQ poisoning. One study found that early, repeated HP combined with HD significantly improved the short-term survival of patients. This combined approach also led to improvements in blood-gas indices, as well as liver and kidney function, compared to conventional treatment with early repeated HP alone [17]. A case series also reported survival in patients who underwent multiple cycles of both HP and HD as part of their treatment regimen [28]. These findings suggest that a combination of HP and HD might offer a synergistic effect in removing PQ and supporting vital organ functions, leading to better patient outcomes. Another prospective controlled study suggested that treatment with continuous veno-venous hemodiafiltration (CVVHDF) and HP improved 90-day survival rates (HR, 0.43; 95% CI, 0.24–0.76; *p* = .004), compared with conventional therapy [29].

Despite its potential benefits, HP has certain limitations and potential complications. A typical HP session might last around 2 h and might not be as efficient in clearing small molecular weight toxins once the toxin has distributed into tissues. HP may also have a limited ability to remove inflammatory factors or maintain the body’s internal environmental balance [30]. Potential complications associated with HP include thrombocytopenia, leukopoenia and hypoglycaemia [31]. Additionally, the effectiveness of HP might be diminished in patients who present with very low initial plasma PQ concentrations, suggesting that its utility might be more pronounced in cases with higher initial toxin levels [32]. Therefore, while HP can be an effective tool for clearing PQ from the bloodstream, its limitations in addressing tissue-bound toxin and the potential for complications need to be carefully considered in the overall management strategy.

## CRRT in PQ poisoning

CRRT is an extracorporeal blood purification method commonly used in intensive care settings to provide continuous removal of solutes and fluid from the blood [33]. In the context of PQ poisoning, CRRT aims to prevent or manage acute kidney injury (AKI), a frequent complication and also to facilitate the removal of PQ from the plasma [34]. The mechanism of action involves the continuous movement of solutes across a semi-permeable membrane. Notably, the convective clearance achieved in CRRT can be advantageous for removing a broader range of molecules, potentially overcoming the limitations of diffusion-based therapies like HD for certain solutes [35]. The continuous nature of CRRT offers sustained removal of PQ, which is particularly important for managing the rebound of the toxin from tissues and provides better control of fluid balance and electrolytes in critically ill patients.

Several clinical studies and case series have explored the effectiveness of CRRT in the management of PQ poisoning. Randomized controlled trials indicated that continuous veno-venous hemofiltration (CVVH) may prolong the survival time of the patients but not reduce mortality [36–38]. But the results need to be interpreted with caution due to the difficulty in conducting randomized trial, small sample size and relatively high risk of bias. One study comparing HP, CRRT and a combination of both (HP+CRRT) found that in patients with plasma PQ levels between 1000 and 5000 ng/mL, CRRT alone showed better outcomes than HP alone, and the combination of HP and CRRT yielded the highest survival rates. While this seems to contradict the primary role of HP, it does not negate the importance of early HP. Rather, it suggests that while early HP is crucial for initial peak toxin reduction within the first 4–6 h, CRRT’s sustained clearance may be more effective at managing the continuous redistribution of PQ from tissues back into the plasma that occurs after the initial peak, particularly in patients with moderate but prolonged toxicity. Specifically, the mortality rate in the CRRT treatment group was 48%, compared to 59.2% in the HP group and 37.9% in the combined treatment group for this specific range of PQ levels [39]. A case report described the successful treatment of severe PQ poisoning using continuous hemodiafiltration (CHDF), a modality within the CRRT spectrum, where the patient recovered without any long-term health issues [40]. Furthermore, a case report highlighted the successful management of a toddler who accidentally ingested PQ, using CRRT along with steroids and antifibrinolytics, including methylprednisolone pulse therapy (20 mg/kg/day) and a continuous N-acetylcysteine infusion (50 mg/kg/h load then 6.25 mg/kg/h), with no evidence of pulmonary fibrosis at a six-month follow-up [41]. These findings collectively suggest that CRRT, especially when integrated into a comprehensive treatment strategy that might include HP, can improve survival outcomes in patients with moderate to severe PQ poisoning. The successful case reports also indicate its potential utility in very severe cases and even in paediatric populations.

Beyond the direct removal of PQ, CRRT plays a crucial role in supporting renal function, which is often compromised in PQ poisoning [42]. By managing AKI, CRRT helps preserve a major route for PQ elimination and prevents secondary complications from uraemia and fluid overload, which are critical for overall patient survival. PQ undergoes redox cycling, generating superoxide anions that lead to lipid peroxidation, mitochondrial dysfunction and cellular damage. This oxidative stress depletes antioxidant defences, including total antioxidant capacity and thiol groups, leading to renal cell apoptosis, inflammation and tubular necrosis [43]. Since the kidneys are a major route for the elimination of PQ, maintaining adequate renal function is essential for the overall management of the poisoning. It is reasonable to apply CRRT for PQ poisoning, given its role in enhancing toxin clearance and mitigating oxidative stress-induced renal injury. Studies also support the use of CRRT in paraquat poisoning [29,40,41]. However, there is a lack of evidence regarding the optimal combination of CRRT with other blood purification modalities.

Regarding the optimal timing and duration of CRRT in PQ poisoning, evidence from the abovementioned studies suggests that it is most effective when initiated in the early stages of poisoning. In one study, CRRT was started within 24 h of PQ ingestion. The typical duration of CRRT in some studies was around 8 to 12 h per day [39]. Among the various modalities of CRRT, CVVH appears to be commonly used in the context of PQ poisoning. One study described a treatment regimen involving HP followed by CVVH [44], while another compared the outcomes of HP alone versus HP combined with CVVH [19]. In the latter study, while the combination therapy prolonged survival time, it did not lead to a statistically significant improvement in overall mortality compared to HP alone. Notably, intensive CVVHDF, which combines HDF and HD, was used successfully in a case of diquat, a herbicide with similar toxicological properties to PQ, poisoning, suggesting its potential applicability in severe PQ poisoning as well [45].

## Hemodiafiltration (HDF) in PQ poisoning

HDF is an extracorporeal renal replacement therapy that combines the principles of HD and hemofiltration [46]. By utilizing both diffusion and convection, HDF aims to remove a broader spectrum of toxins from the blood, encompassing both small and medium-sized molecules, as well as providing effective fluid and electrolyte management [46]. In the context of PQ poisoning, the dual mechanism of HDF theoretically offers a potential advantage in clearing PQ, which has a relatively low molecular weight, including fluid overload and electrolyte imbalances that can arise, particularly with kidney injury.

The available evidence specifically on the use of HDF in PQ poisoning is relatively limited compared to HP and HD. However, a case report documented the successful treatment of severe PQ poisoning using CHDF [40]. In this case, the patient, who had ingested a lethal dose and developed renal dysfunction, underwent CHDF and subsequently recovered without any long-term health issues, suggesting that HDF can be a viable treatment option in severe cases. One study that compared different extracorporeal therapies mentioned that hemodiafiltration, with a clearance rate less efficient than HP (116 ± 32 mL/min) in terms of PQ removal rate but allows for a longer duration of continuous treatment, which could be beneficial in sustaining toxin removal over several days [47]. Interestingly, intensive CVVHDF was used successfully in a case of diquat poisoning, in a patient who developed rhabdomyolysis and shock [45]. This regimen involved ‘intensive HP combined with continuous renal replacement therapy’. However, the exact HP protocol was not reported. This successful outcome in a related poisoning scenario suggests that HDF might have a role to play in managing severe PQ poisoning as well, especially when prolonged treatment is required. The successful cases of CHDF in severe PQ poisoning demonstrates that this modality can effectively contribute to PQ clearance and lead to a positive patient outcome.

Despite these promising findings, the evidence base for the use of HDF in PQ poisoning remains limited, with a paucity of dedicated clinical studies compared to HP and HDF. Therefore, further research is needed to more clearly define its role, optimal application strategies and its comparative effectiveness against other extracorporeal therapies in the management of PQ poisoning.

## Haemodialysis (HD) in PQ poisoning

HD is a widely available extracorporeal therapy that removes waste products and excess fluid from the blood using diffusion across a semi-permeable membrane [48]. Historically, HD has been employed in the management of PQ poisoning with the dual aim of enhancing the elimination of the toxin from the bloodstream and addressing the AKI that frequently develops as a complication [49–51]. Given its widespread availability and established role in managing kidney failure, HD has been a natural consideration for PQ poisoning, especially in patients who develop renal impairment. However, the effectiveness of HD in directly removing PQ and improving overall survival has been a subject of ongoing debate and investigation [21,52,53].

A systematic review and meta-analysis that specifically examined the efficacy of HD on PQ poisoning mortality concluded that, based on the available evidence, HD did not significantly affect the survival of patients [21]. In fact, this meta-analysis, which included several studies and a combined total of 203 patients, found a statistically significant trend towards higher mortality in the group that underwent HD compared to those who did not (OR 2.84; 95% confidence interval [CI]: 1.22–6.64; *p* = .02). This negative association does not necessarily mean HD is inherently harmful; rather, it may be due to confounding by indication. Patients who receive HD are often more severely ill, presenting with significant AKI, so the observed increased mortality may reflect the severity of the underlying poisoning rather than a detrimental effect of the therapy itself. While some individual studies have reported that HD might reduce mortality in certain subgroups of patients, others have found little to no effect on patient recovery. This meta-analytic evidence strongly suggests that HD as a primary treatment for PQ poisoning is not effective in improving survival outcomes and might even be associated with worse results. This contradicts earlier hopes and practices and necessitates a re-evaluation of the role of HD in the management of this severe poisoning.

Despite the findings of the meta-analysis, mortality rates in many studies involving HD for PQ poisoning have remained high. One study specifically noted that HD did not reduce mortality in their cohort, potentially due to delayed presentation of patients for treatment [25]. Nevertheless, HD continues to be used in many centres as a means of attempting to excrete PQ and, more importantly, to manage the AKI [54]. A case series suggested a potential correlation between earlier initiation of HD and better survival rates, with survivors in their study receiving dialysis on average earlier than non-survivors [55]. This observation hints that while HD might not directly counteract the primary toxic effects of PQ, very early initiation could potentially play a supportive role, possibly by managing renal complications more effectively or by removing a small amount of circulating toxin before it causes extensive tissue damage.

Evidence indicates that even when delays in setting up HD are minimized, the elimination of PQ achieved by this method might be limited because the toxin rapidly disappears from the plasma into tissues [56]. Studies have shown that the actual amounts of PQ removed by HD over typical 6 to 8-h sessions are often negligible in most cases [49]. A case series from India suggested that better survival was observed when HD was started earlier (mean 1.13 days in survivors versus 2 days in non-survivors), although this might reflect better management of renal failure rather than enhanced PQ removal [55]. The consensus emerging from the literature is that the quantities of PQ recovered by HD are generally insignificant in relation to the total body burden. HD is therefore unlikely to prevent death in patients with plasma PQ concentrations significantly above the prognostic threshold for survival. In fact, as highlighted by the meta-analysis, some evidence even points towards a potential for higher mortality in patients treated with HD [21]. These findings suggest that while HD remains an important tool for managing the renal complications of PQ poisoning, it should not be considered a primary therapy for the removal of the toxin itself. Its role is likely best limited to supportive care, particularly in addressing AKI and severe acid-base disturbances that may arise.

## Therapeutic plasma exchange (TPE) in PQ poisoning

TPE, also known as plasmapheresis, is an extracorporeal procedure that involves removing a patient’s plasma, which may contain harmful substances like toxins or antibodies and replacing it with fresh frozen plasma or albumin [57]. In the context of PQ poisoning, TPE has been considered as a potential therapy to rapidly lower the levels of circulating PQ or associated toxic mediators, particularly those that might be protein-bound or have a limited volume of distribution [58,59]. The rationale behind using TPE is that by removing plasma, it might be possible to reduce the systemic concentration of the toxin before it causes irreversible damage, especially in severe cases.

While randomized controlled trials specifically investigating the efficacy of TPE in PQ poisoning are lacking, several case reports and a few case series have suggested its potential value in managing this condition [60]. It is important to emphasize that the current evidence for TPE is limited to case reports and small series, which constitutes a low level of evidence (Level 4 according to the Oxford Centre for Evidence-Based Medicine). Therefore, while these findings are hypothesis-generating, definitive conclusions on TPE’s efficacy cannot be drawn without higher-quality evidence. Reports in the literature have indicated the utility of TPE in treating various drug overdoses, suggesting that it can be an effective method for rapidly removing toxic substances from the bloodstream [60]. One study reported experience of treating intoxication with TPE, including one case of PQ poisoning, where TPE was performed twice at day 3 and day 10 of admission for the PQ poisoning patients, while the patient was dead at the third day after second TPE session [59]. These findings, although preliminary, hint that TPE might contribute to improved survival or reduced morbidity in patients with PQ poisoning. A prospective longitudinal study focusing on non-neurological conditions included a cohort of 19 patients with PQ poisoning who were treated with TPE, with a promising 79% of these patients showing clinical improvement following the procedure [61], indicating a potentially positive impact on clinical outcomes. Additionally, one study mentioned that plasma perfusion therapies, which are related to plasma exchange, have shown promise in aiding the clearance of PQ and could be a valuable therapeutic tool in patients with acute PQ intoxication [62]. These initial findings from case-based studies and small series suggest that TPE might offer a beneficial role in the management of PQ poisoning, particularly when initiated early in the course of the illness. However, the absence of large-scale, controlled trials necessitates caution in drawing definitive conclusions about its overall efficacy.

TPE is generally considered for severe cases of intoxication where the prognosis is poor, or when a patient’s condition is worsening despite conventional therapies. Guidelines for therapeutic apheresis suggest that urgent plasma exchange should be initiated as early as possible, ideally within 24 to 36 h of diagnosis, in life-threatening situations where there are no other valid therapeutic alternatives [58,63]. This recommendation underscores the potential importance of early intervention with TPE in severe PQ poisoning.

Despite these promising signals, there are several limitations and considerations associated with the use of TPE in PQ poisoning. A significant limitation is the lack of robust evidence from randomized controlled trials that specifically evaluate its efficacy in this setting. Additionally, TPE might be less effective in removing small, nonprotein-bound molecules, which could be a factor in the later stages of PQ poisoning when the toxin is less likely to be bound to plasma proteins. TPE is also an expensive procedure, and it is associated with potential complications such as hypotension, allergic reactions, bleeding and sepsis [64]. Therefore, the decision to use TPE in PQ poisoning requires a careful consideration of the potential benefits against these risks and limitations, particularly in the absence of strong evidence from large-scale clinical trials.

## Comparative analysis of extracorporeal therapies

When comparing the different extracorporeal therapies for PQ poisoning, several key distinctions and potential roles emerge from the current evidence. HP is generally considered more effective than HD for the direct clearance of PQ from the bloodstream. While HP might offer more rapid toxin removal in the short term, HDF has the advantage of allowing for longer durations of continuous treatment, which could be beneficial for sustained PQ elimination. In contrast, HD as a standalone therapy for PQ poisoning seems to have limited benefit and might even be associated with worse outcomes. The comparison of clearance rates indicates that while HDF might not be as rapid as HP in removing PQ from the blood, its capacity for extended continuous removal could potentially compensate for a slightly lower per-minute efficiency. When compared to other extracorporeal therapies, HP is generally regarded as the most effective for rapid, short-term PQ clearance. TPE offers a different mechanism by removing the entire plasma component, which may be beneficial for toxins that are protein-bound or to clear inflammatory mediators, but its efficacy is supported by lower-quality evidence and it comes with significant risks and costs.

**Figure 2.**
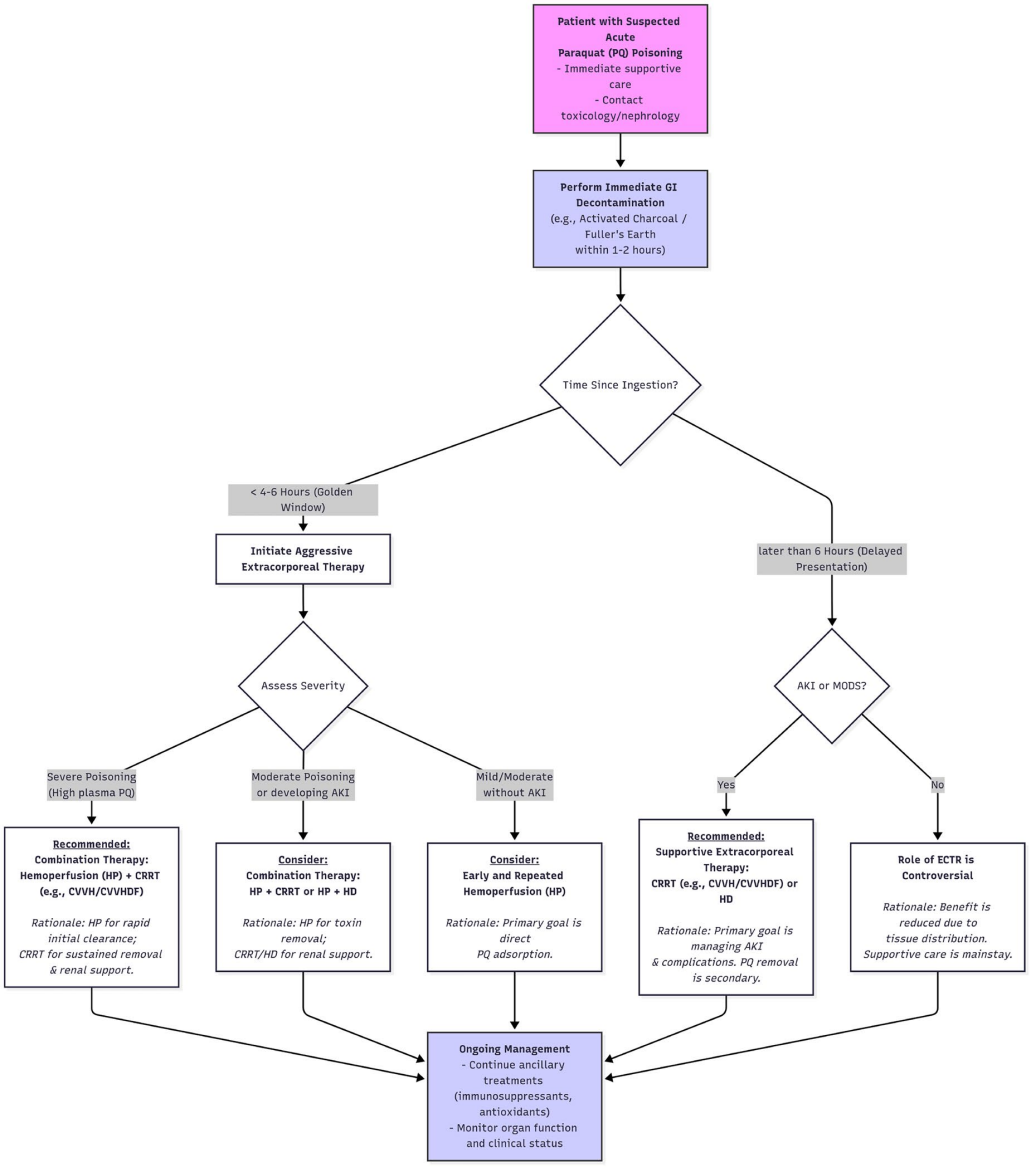
Clinical treatment algorithm. AKI: acute kidney injury; CRRT: continuous renal replacement therapy; CVVH: continuous veno-venous hemofiltration; CVVHDF: continuous veno-venous hemodiafiltration; ECTR: extracorporeal therapy; HD: haemodialysis; HP: hemoperfusion; HDF: hemodiafiltration

Several factors appear to influence the choice of extracorporeal therapy in PQ poisoning. The timing of intervention is crucial for all modalities, with earlier treatment generally associated with better outcomes, as it aims to remove the toxin before extensive tissue damage occurs [65]. The severity of poisoning, often guided by prognostic tools like the Severity Index of Paraquat Poisoning (SIPP) or plasma PQ concentrations, might also guide the intensity and type of therapy chosen [66,67]. The presence of AKI, a common complication, might favour the use of CRRT or prolonged HDF due to their ability to provide continuous renal support. Practical considerations such as the availability of specific therapies and the expertise of the medical team also play a significant role in clinical decision-making (Figure 2). Given these various factors, a tailored approach to selecting the most appropriate extracorporeal therapy, based on an individual patient’s clinical presentation and circumstances, is likely necessary.

The potential for combination therapies involving different extracorporeal modalities is also an important consideration (Table 2). Combining HP with HD has shown improved survival rates and better organ function in some studies [17]. The favourable outcomes observed with CVVHDF suggest that CVVHDF following HP may represent an optimal therapeutic approach. Nevertheless, direct evidence supporting this strategy remains limited. The successful use of intensive HP combined with CRRT in a case of diquat poisoning suggests that this approach might also be beneficial in severe PQ poisoning [45]. These findings indicate that combination therapies, particularly those that leverage the strengths of different extracorporeal techniques and address multiple aspects of PQ toxicity (e.g. rapid toxin removal *via* HP coupled with sustained renal support *via* CRRT or HD), hold promise and warrant further investigation.

**Table 2.** Evidence for combination extracorporeal therapies.

Combination therapy	Evidence of benefit	Possible synergistic effects
Hemoperfusion (HP) + haemodialysis (HD)	Improved blood-gas indices, liver and kidney function and short-term survival. Higher three-month survival rate observed.	HP for rapid toxin removal, HD for managing renal failure and small molecular toxins.
Hemoperfusion (HP) + continuous renal replacement therapy (CRRT)	Highest survival rates in patients with plasma paraquat levels between 1000 and 5000 ng/mL. Reduced fatality rates in early stages.	HP for initial toxin clearance, CRRT for sustained removal and renal support.
Hemoperfusion (HP) followed by continuous veno-venous hemofiltration (CVVH)	Prolonged survival time, prevented early death from circulatory collapse (though no overall mortality improvement in one study).	HP for initial removal, CVVH for continuous filtration and cytokine removal.

## Conclusion and recommendations

The current evidence regarding the use of extracorporeal therapies in PQ poisoning suggests a complex and nuanced picture. HP appears to be a promising modality for reducing mortality, especially when initiated early after ingestion and potentially when combined with other treatments like HD. CRRT, particularly in the form of CVVH, shows benefits in patients with moderate to severe poisoning, especially when used in conjunction with HP and offers crucial support for renal function. The evidence for HDF is limited but suggests potential utility in severe cases and for prolonged treatment durations. In contrast, HD as a standalone treatment for PQ poisoning does not appear to improve survival and might even be detrimental; its primary role is supportive, specifically for managing severe AKI (e.g. stage 3), uncontrolled hyperkalaemia, or severe metabolic acidosis (Table 3). TPE shows potential based on case reports and a small case series, especially if administered early in severe cases, but lacks strong support from randomized controlled trials.

**Table 3.** Correlation of PQ ingestion, plasma levels and recommended therapy.

Severity category	Plasma PQ level (approx. at 4–6h)	Clinical manifestations	Recommended extracorporeal therapy
Mild	<1000 ng/mL	Often asymptomatic or mild GI upset; high survival	Observation; supportive care; consider single HP session if levels are borderline
Moderate to severe	1000–5000 ng/mL	GI corrosive injury, AKI, progressive pulmonary fibrosis	Emergency HP + CRRT (highest survival in this range)
Fulminant	>5000 ng/mL	Multi-organ failure, circulatory collapse, death within 24–72h	Immediate aggressive HP + CRRT (may only prolong survival/palliative)

Based on the current evidence, the following recommendations for clinical practice can be considered: Early HP, ideally within the first 4 to 6 h of PQ ingestion, should be strongly considered, particularly in cases with a high likelihood of severe poisoning based on the amount ingested or initial clinical assessment. Combining HP with CRRT (especially CVVH) might offer the best outcomes in patients with moderate to severe PQ poisoning, addressing both toxin removal and renal support. HD should not be the primary extracorporeal therapy aimed at removing PQ but remains an important tool for managing AKI and severe electrolyte or acid-base imbalances. TPE could be considered in severe cases, particularly early after ingestion, as suggested by case-based evidence, but its use should be carefully weighed against the lack of strong clinical trial data and the potential risks associated with the procedure. Ultimately, treatment protocols for PQ poisoning should be individualized, taking into account the time elapsed since ingestion, the estimated severity of poisoning (ideally using plasma PQ levels if available) [44] and the patient’s overall organ function status.

There are several gaps in our knowledge regarding the optimal use of extracorporeal therapies in PQ poisoning, highlighting important areas for future research. More randomized controlled trials are urgently needed to definitively establish the efficacy of different extracorporeal therapies, both as single modalities and in combination, in improving survival and other clinically relevant outcomes. The optimal timing, duration and specific protocols for each therapy need further investigation to maximize their potential benefits. The role of HDF in this context requires more dedicated studies to determine its effectiveness and ideal application. Future research should also explore the potential of emerging extracorporeal technologies. For instance, the Molecular Adsorbent Recirculating System (MARS), which is designed to remove protein-bound and water-insoluble toxins, could theoretically be beneficial in paraquat-induced hepatic failure. Recent studies have demonstrated the hepatoprotective role of MARS in acute liver injury and toxin-related hepatic failure [68], suggesting that its application in paraquat poisoning warrants further investigation.

## Data Availability

Data sharing is not applicable to this article as no new data were created or analysed in this study.
